# Chemical Environment and Temperature Effects on the Formation and Destruction of C_3_O_2_ in Cosmic-Ray-Processed Ices

**DOI:** 10.1021/acsomega.5c11198

**Published:** 2026-02-18

**Authors:** Sergio Pilling, Felipe Fantuzzi, Diana P. P. Andrade, Leonardo Moraes

**Affiliations:** † Instituto de Pesquisa e Desenvolvimento, Universidade do Vale do Paraíba (UNIVAP), São José dos Campos 12244-000, São Paulo, Brazil; ‡ School of Natural Sciences, University of Kent, Park Wood Rd., Canterbury CT2 7NH, U.K.; § Observatório do Valongo, Universidade do Federal do Rio de Janeiro (UFRJ), Rio de Janeiro 20080-090, Rio de Janeiro, Brazil

## Abstract

Astrophysical ices composed of CO and CO_2_ undergo complex radiation-driven chemistry, producing reactive species with potential prebiotic relevance. Using the PROCODA kinetic model (642 coupled reactions, 18 tracked species) combined with ion irradiation data, we investigate the main formation and destruction pathways of carbon suboxide (C_3_O_2_) in CO-, CO_2_-, and mixed CO/CO_2_-rich ices. A clear two-regime picture emerges. At early fluence, chemistry is matrix-controlled: in pure CO ice, C_3_O_2_ forms mainly via CO + C_2_O → C_3_O_2_, whereas in pure CO_2_ ice it proceeds via CO_2_ + C_2_O_2_ → O_2_ + C_3_O_2_; mixed ices retain CO-involving channels. At chemical equilibrium, routes shift as accumulated intermediates take over: in CO ice, C_3_ + CO_2_ → C + C_3_O_2_ dominates, while in CO_2_ ice, CO + C_2_O_2_ → O + C_3_O_2_ prevails. Destruction is likewise environment-sensitive: C_3_O_2_ + *R* → CO + C_2_O leads in CO ice, versus C_3_O_2_ + *R* → C + C_2_O_2_ in CO_2_ ice (*R* denotes radiation-induced processes). Raising the temperature from 10 to 20 K enhances bimolecular channels through greater molecular mobility, while leaving radiation-driven pathways largely unaffected. Using C_3_O_2_ as a prototype, this study provides pathway maps that link composition, temperature, and irradiation history, offering new constraints for astrochemical models and for interpreting JWST and ALMA observations.

## Graphical abstract

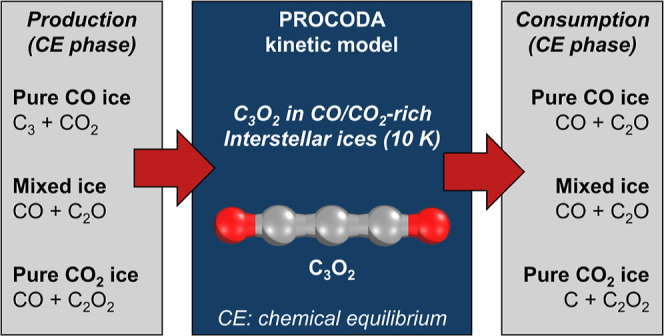

## Introduction

Astrophysical ices in dense molecular clouds and protoplanetary disks are largely composed of simple volatiles such as CO, CO_2_, H_2_O, CH_4_, and NH_3_. Under ionizing radiation and cosmic-ray bombardment, these ices undergo complex chemical transformations that progressively yield larger and more complex species.^1,2^ At fixed temperature, prolonged irradiation drives the system toward a chemical equilibrium (CE) phase in which formation and destruction pathways balance.^3,4^ During this progression, the evolving molecular environment reshapes reaction kinetics and desorption processes.^5^

The influence of the local environment on reaction dynamics is well established. Alves et al.^6^ demonstrated that dielectric properties of the matrix strongly modulate kinetics: polar H_2_O-rich ices stabilize intermediates and transition states, raising activation barriers and slowing reaction rates, whereas ices with low dielectric constant, such as CO-rich ices, facilitate tunneling and lower barriers, enhancing reactivity. Such physicochemical effects determine not only kinetics and thermodynamics but also infrared band profiles used in astronomical quantification.^5–12^ Accurate astrochemical models must therefore incorporate matrix effects when predicting abundances of reactive species and prebiotic precursors.

Among the radiation products of CO- and CO_2_-rich ices, carbon suboxide (C_3_O_2_) is of particular interest. With enhanced stability among odd-numbered polycarbon oxides,^13^ it is efficiently produced by UV photons, energetic ions, and keV electrons.^4,14–21^ Upon irradiation, its polymerization followed by ice warming produces low-volatility reddish-brown solids, consistent with the dark albedo observed on cometary surfaces such as Halley.^22,23^ Despite strong infrared bands, including ν_3_ near 2258 cm^–1^, C_3_O_2_ has not been directly identified in cometary comae,^24^ and its quasi-linear structure complicates radio detection. Laboratory studies, however, consistently reproduce its formation under irradiation, while detections of related oxides (C_3_O_2_, C_2_O, C_3_O) toward Elias 18,^25^ a young Class I object which possesses a dust- and ice-rich envelope, and in dense molecular clouds^26^ support its astrophysical relevance. Its reactive carbonyl groups enable further radical recombination and polymerization, suggesting roles in pathways toward complex organics and prebiotic species.^26,27^

To investigate how environment and temperature influence the kinetics of irradiated astrophysical ices, we selected the radiolytic product C_3_O_2_ as a prototype molecule. We employed the PROCODA kinetic model, which integrates 642 coupled reactions involving 18 species, to revisit previously studied CO/CO_2_ mixtures^20^ and pure CO ices at multiple temperatures^19^ irradiated by cosmic-ray analogs. By systematically mapping dominant formation and destruction pathways, we evaluated how matrix composition and temperature modulate effective rate coefficients and equilibrium abundances. The central aim of this work is to characterize the preferential reaction routes of C_3_O_2_ as a function of the initial CO/CO_2_ ratio and ice temperature, and to assess the implications of environment-driven variations in reaction kinetics.^6,28^ Ultimately, elucidating these pathways is essential for refining astrochemical models and for identifying the molecular mechanisms that may connect simple ices to prebiotic chemistry, thereby advancing our understanding of both astrochemistry and astrobiology.

The Methods section outlines the experimental data and computational methodology employed in this study, including the determination of effective rate coefficients (ERCs) and the treatment of chemical equilibrium in irradiated ices. The Results and Discussion section presents the preferential formation and destruction pathways of C_3_O_2_ obtained from PROCODA simulations, together with the associated astrochemical implications. Finally, the Conclusions section summarizes the key findings of this work.

## Methods

In this study, we revisited the PROCODA kinetic model outputs from previous investigations on mixed CO/CO_2_ ices (pure CO ice (EXP1); mixed CO/CO_2_ (3.9:1) ice (EXP4); pure CO_2_ ice (EXP7)) irradiated at 10 K using 95.2 MeV ^136^Xe^23+^ ions,^20^ as well as pure CO ices at three different temperatures (10 K, 15 and 20 K) irradiated under the same conditions.^19^

Briefly, the experiments were performed at the ultrahigh vacuum (UHV) chamber IGLIAS (Irradiation de GLaces d’Intérêt Astrophysique) as detailed by Augé et al.,^29^ in conjunction with the IRRSUD beamline at the Grand Accélérateur National d’Ions Lourds (GANIL) in Caen, France. Additional details on the experimental data are provided in refs 19,20 . In summary, the gas samples were deposited in situ onto a clean, cryogenic ZnSe substrate and irradiated with 95.2 MeV ^136^Xe^23+^ ions. The chemical evolution of the ices was then monitored in situ by Fourier-transform infrared (FTIR) spectroscopy over the 4000–650 cm^–1^ range with 1 cm^–1^ resolution. During the irradiation stage, the ion flux was maintained at 5 × 10^8^ ions cm^–2^ s^–1^, with total fluences reaching 3 × 10^12^ ions cm^–2^.

It is important to clarify for readers an experimental limitation concerning the CO/CO_2_ (3.9:1) mixture used in ref 20. At the time of those experiments, achieving an exact 1:1 solid-phase mixture was not experimentally feasible. Although the gas-line mixture delivered nominally equal partial pressures for both species, the resulting ice composition deviated from 1:1 due to two well-known experimental constraints: (i) CO and CO_2_ have different flow dynamics through the capillaries into the vacuum chamber, and (ii) they possess different sticking coefficients on the cryogenic substrate during film growth. These factors prevent a precise 1:1 CO/CO_2_ ratio from being reliably deposited in the solid phase.

In the temperature-dependent experiments on pure CO ices (ref 19), the ices were first deposited at 10 K and then slowly warmed to the target irradiation temperature (10, 15, or 20 K). During this warming step, structural rearrangement and annealing of radiation-induced defects are expected, a behavior consistent with typical astrophysical environments. Importantly, once irradiation began, the sample temperature was held constant by the high-power closed-cycle helium cryostat. All chemical evolution was therefore mapped under strictly isothermal conditions (10, 15, or 20 K, depending on the experiment) and compared directly with the corresponding unirradiated ice.

As discussed in refs 19,20 , the selected projectile (95.2 MeV ^136^Xe^23+^) is a representative swift heavy ion, a typical high-mass component of the cosmic-rays. Although not the most abundant cosmic-ray species, such ions are astrophysically relevant because their high charge state during penetration enables extremely efficient electronic energy deposition via cascades of secondary electrons. These cascades drive excitation, ionization, dissociation, and a variety of physicochemical processes, including reactions and desorption, at levels far exceeding those produced by abundant cosmic-ray protons.

Our primary objective in this work is to analyze how chemical environment and temperature modulate the formation and destruction of frozen molecules, using C_3_O_2_ as a prototype, in cosmic-ray-processed ices containing CO and/or CO_2_. To achieve this, we identify and characterize the dominant reaction pathways responsible for C_3_O_2_ production and loss, evaluate their dependence on the initial CO/CO_2_ ratio, examine temperature-driven variations in pure CO ices, and discuss how the surrounding chemical environment influences reaction kinetics within astrophysical ices.

The PROCODA code was employed to model the kinetic evolution of 18 molecular species (C, O, C_2_, CO, O_2_, C_3_, C_2_O, CO_2_, O_3_, C_3_O, C_2_O_2_, CO_3_, C_4_O, C_3_O_2_, C_2_O_3_, C_5_O, C_4_O_2_, C_5_O_2_) within the ice, solving a system of 642 coupled chemical reactions. eq 1 describes the time evolution of the column density, *N*_*i*_ (molecules cm^–2^), of species *i*, considered in the PROCODA code

1 $$\frac{dN_{i}}{dt}=-DES_{i}\left(t\right)-\sum_{d1}k_{d1}N_{i}\left(t\right)-\sum_{d2}k_{d2}\frac{N_{i}\left(t\right)N_{a}\left(t\right)}{L}+\sum_{p1}k_{p1}N_{a}\left(t\right)+\sum_{p2}k_{p2}\frac{N_{a}\left(t\right)N_{b}\left(t\right)}{L}$$

where *dN*_*i*_/*dt* is given in molecules cm^–2^ s^–1^, and *L* is the sample thickness (cm). The rate constants *k*_*d*1_ and *k*_*p*1_ refer to direct (single-body or radiation-induced) destruction and production processes induced by radiation (units of s^–1^), whereas *k*_*d*2_ and *k*_*p*2_ describes the bimolecular rate coefficients for collision processes (units of cm^3^ molecule^–1^ s^–1^). The term *DES*_*i*_(*t*) quantifies desorption (molecules cm^–2^ s^–1^) into the gas phase, whereas *N*_*a*_ and *N*_*b*_ are the column densities of species *a* and *b*, respectively. Here, the values *k*_*d*1_, *k*_*d*2_, *k*_*p*1_, and *k*_*p*2_ are also named as effective rate coefficients (ERCs). Further details and the complete set of coupled equations can be obtained in Pilling et al.^20^

The model incorporates species observed in the experiments (e.g., CO, CO_2_, C_3_O_2_, O_3_, and C_2_O), along with additional species expected but not observed (e.g., C_2_O_2_, C_4_O, C_5_O). The ERCs and desorption rates were derived by minimizing a score function that considers mass conservation, chemical equilibrium constraints, and infrared spectral fits (see details in ref 20).

It is important to note that the original models discussed by Pilling et al.^19^ for pure CO ices at three temperatures considered only 156 reactions. In contrast, a new set of calculations using the PROCODA code with 642 reactions (also accounting for CO_3_ as an observed species in negligible amounts) was performed on this data set to ensure that the analysis of the dominant reaction pathways involved in the formation and destruction of C_3_O_2_ remains consistent with that conducted for mixed ices (see also ref 20). Detailed results for the pure CO ice at 10 K under the same irradiation conditions are provided in Pilling et al.^20^ The model parameters shown in the figure headers are discussed in detail in the cited references.

For clarity, Figure 1 shows the comparison between PROCODA kinetic simulations and experimental data for CO-rich and CO/CO_2_-mixed ices irradiated with 95.2 MeV ^136^Xe^23+^ ions at 10 K. The colored symbols denote the experimental results, while solid lines represent the best-fit models. The bold-dashed blue line represents the modeled summed desorption column density at a given time (proportional to the amount of molecules that goes to gas-phase during ice irradiation). It is important to emphasize that several intermediates and radicals appear in the model because the ice receives a continuous influx of energy from cosmic-ray analogs. Without sustained energy deposition, many of these transient species would rapidly vanish, as they would react toward more stable products without being continuously regenerated (see additional details at refs 19,20 ).

**Figure 1:**
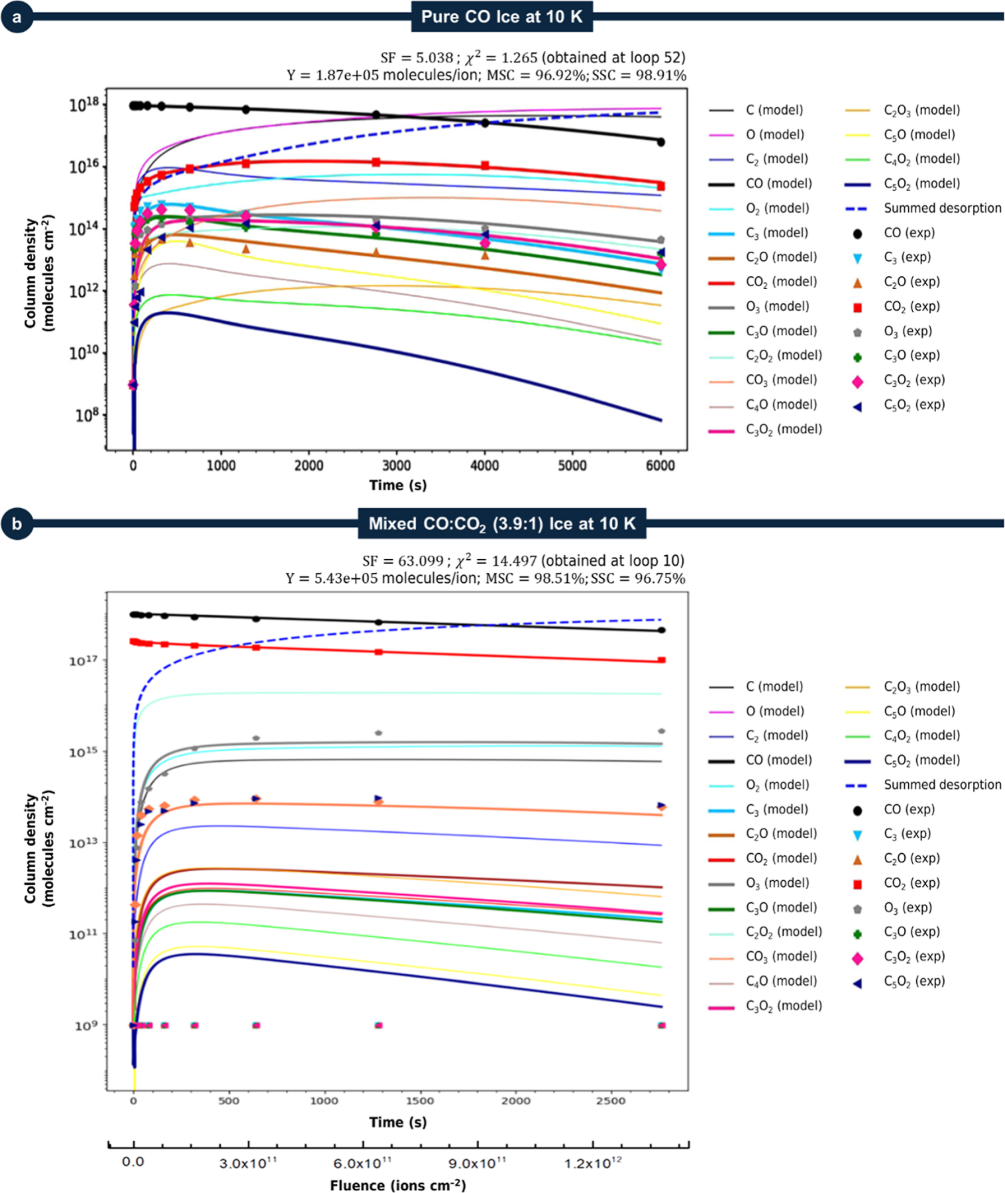
Best-fit PROCODA kinetic models compared with experimental data for astrophysical ice analogs irradiated with 95.2 MeV ^136^Xe^23+^ ions at 10 K. Colored symbols denote experimental results and solid lines represent model outputs for (a) pure CO ice and (b) mixed CO/CO_2_ (3.9:1) ice. Chemical equilibrium appears as a sloped plateau at high fluences due to desorption, with desorption yields (*Y*, molecules/ion) given in the header. See details in the text.

Important model output parameters are displayed in the header including the total desorption yield (*Y*) in units of molecules/ion. The experimental error and model uncertainties were both estimated to lie below 20% (see also ref 30). In pure CO ice (Figure 1a and ref 19), species abundances increase rapidly at low fluences before approaching chemical equilibrium. This equilibrium is expressed as a quasi-stationary plateau with a slow decrease due to desorption. In the mixed CO/CO_2_ (3.9:1) ice (Figure 1b; Pilling et al., ref 20), similar behavior is observed but additional chemical routes from CO_2_ modify the relative abundances of several products. At high radiation fluences, the balance between formation and destruction pathways defines the steady-state composition. The close agreement between model and experiment highlights the reliability of PROCODA in describing environment-dependent chemistry in irradiated astrophysical ices.

As previously discussed (see refs 19, 20 and 30 ), the PROCODA kinetic model is based on the hypothesis that chemical equilibrium (CE) is reached after the ice is exposed to higher fluence. In the CE phase, the system presents a balance between formation and destruction reactions, which occur at rates determined by ERCs. It is worth noting that sputtering or desorption processes continue during the CE phase, gradually decreasing the abundances of all species in the ice while enhancing the gas-phase abundances of radiolysis products in its vicinity. The model further assumes that solid-phase reactions under irradiation can differ from their gas-phase counterparts because matrix effects in the ice modify both the thermodynamics (reaction energetics) and the kinetics (activation barriers).

The methodology employed in this work confers several advantages for astrochemical modeling. Application of PROCODA enabled the derivation of ERCs for numerous pathways in irradiated ices, yielding a quantitative characterization of molecular evolution under space-relevant conditions. The ability to model the interplay between competing reactions provides, for example, a detailed analysis of how parent-ice composition and temperature influence the dominant formation and destruction pathways of specific species, such as C_3_O_2_. Moreover, this approach enables the refinement of astrochemical models by incorporating experimentally constrained reaction networks, thereby reducing uncertainties in theoretical predictions.

In spite of the valuable results provided by PROCODA, including maps of the chemical evolution of observed and predicted species within the ice and estimates of ERCs and desorption parameters, the computational approach also has limitations. For example, the code relies on a large, predefined reaction network that cannot encompass all possible chemical pathways. In addition, ionic species were not explicitly included in the reaction set, and electron-driven channels were not treated directly. While cosmic-ray irradiation generates a cascade of secondary electrons that drive molecular dissociation and recombination, we hypothesize that rapid electron–ion recombination leads to near-instant neutralization, after which the resulting neutrals react. Accordingly, the model represents irradiation chemistry through effective reaction routes parametrized by the calculated ERCs. Additionally, although the model incorporates solid-phase reaction kinetics, it does not fully capture irradiation-induced structural evolution of the ice matrix, which can modify reaction rates as exposure progresses (see refs 31–34 ). Despite these limitations, the systematic derivation of ERCs in coupled ice systems marks a significant step toward more accurate astrochemical models with direct relevance to future JWST and ALMA observations of interstellar and planetary ices.

The full set of equations employed, along with the best-fit models describing the chemical evolution of the studied ices, is presented in detail in Pilling et al.^19,20^

## Results and Discussion

### Influence of Initial Ice Composition in Reaction Routes of C_3_O_2_ during Irradiation by Cosmic Rays

Figure 2 compiles the time-dependent differential production and consumption rates of C_3_O_2_, divided by initial ice column density, in the three ice systems investigated—pure CO (Figure 2a,b), a mixed CO/CO_2_ (3.9:1) ice (Figure 2c,d), and pure CO_2_ (Figure 2e,f)—as predicted by the PROCODA kinetic model at different exposures times *t* = 7 s (small fluence), 500, 1380, and 2760 s (large fluence, within the chemical equilibrium phase). These times were selected to capture both the early irradiation regime (small fluence) and the late-irradiation regime (CE phase, typically *t* > 1500 s in such experiments). For each ice system, the seven dominant reaction pathways responsible for the production and consumption of C_3_O_2_ are shown and labeled (e.g., r78, r146). The corresponding reactions are listed in Table 1, together with approximate gas-phase reaction enthalpies for comparison.

**Figure 2:**
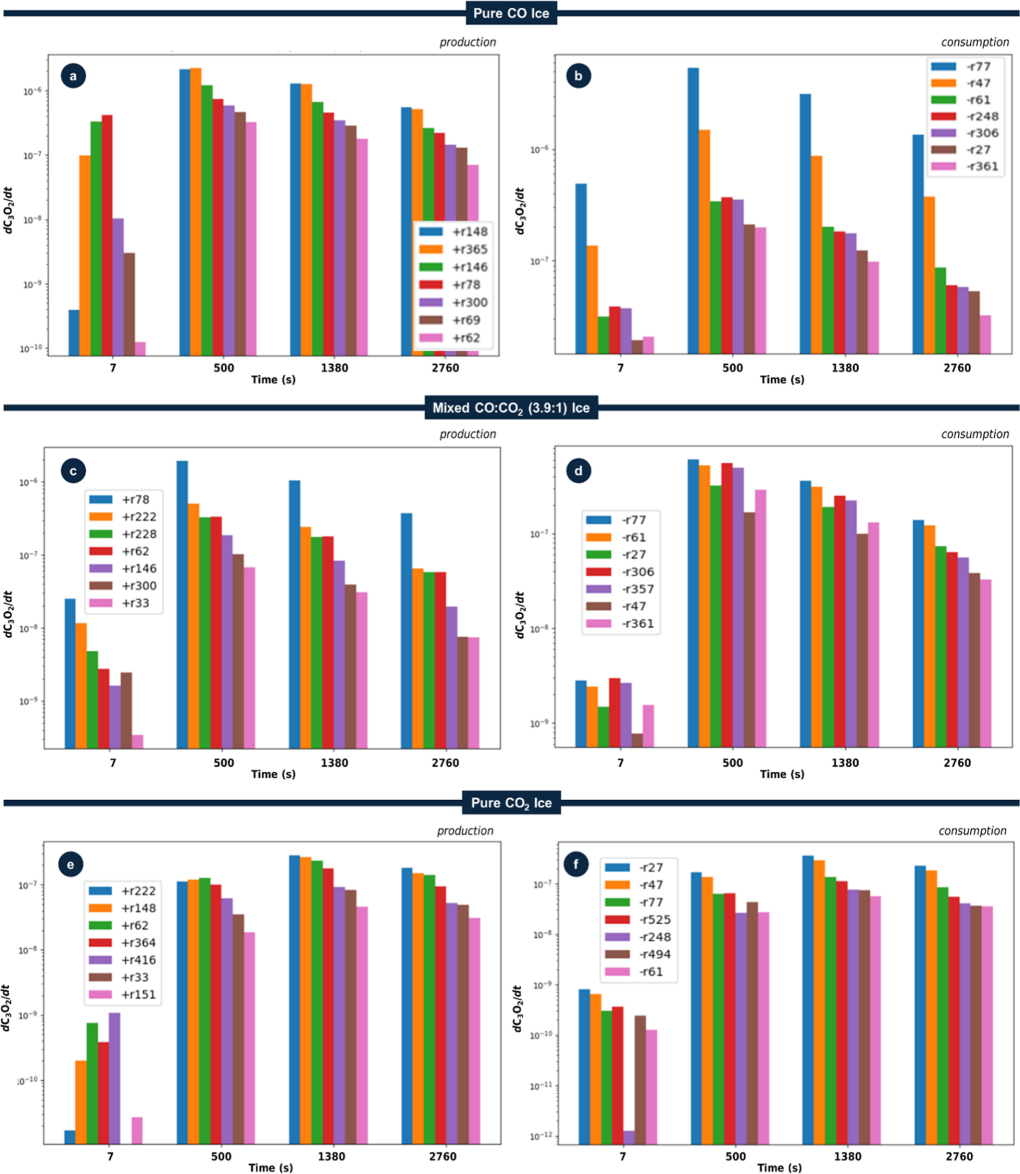
Evolution of the contributions of the seven dominant reaction pathways to the production (a,c and e) and consumption (b,d and f) of C_3_O_2_ in (a,b) pure CO ice, (c,d) mixed CO/CO_2_ (3.9:1) ice, and (e,f) pure CO_2_ ice, all irradiated by cosmic rays at 10 K. The *y*-axis shows the differential production/consumption rate (divided by the largest column density of the parent species at time = 0 s) evaluated at the times indicated on the *x*-axis. Times >1200 s correspond to the chemical-equilibrium (CE) phase. Reaction labels use a sign convention in which + r*n* denotes a production route (e.g., + r148, + r78, + r222) and −r*n* denotes a consumption route (e.g., −r77, −r27). See Table 1 and the main text for details.

**Table 1:** Main Production and Consumption Routes for C_3_O_2_ and Their Corresponding ERCs Used in the Model (Corresponding to Figure 2) for Ices at 10 K

main C_3_O_2_ production reaction routes^a^		pure CO ice (10 K)^b^	CO/CO_2_ (3.9:1) ice (10 K)^b^	pure CO_2_ ice (10 K)^b^
r33	C_4_O_2_ + *R* → C + C_3_O_2_ (Δ*H*_*R*,gas,0K_ = 455.6)	5.94 × 10^–3^	4.84 × 10^–2^	7.15 × 10^–1^
r62	C_2_ + CO_2_ → C_3_O_2_ (−529.23)	1.38 × 10^–23^	5.08 × 10^–24^	1.68 × 10^–24^
r69	C_5_O_2_ + *R* → C_2_ + C_3_O_2_ (566.3)	3.56 × 10^–2^	5.10 × 10^–1^	3.11 × 10^–1^
r78	CO + C_2_O → C_3_O_2_ (−365.3)	1.00 × 10^–24^	5.68 × 10^–23^	1.30 × 10^–23^
r146	CO + C_3_O → C + C_3_O_2_ (393.2)	4.15 × 10^–25^	1.73 × 10^–23^	1.21 × 10^–24^
r148	C_3_ + CO_2_ → C + C_3_O_2_ (187.8)	3.11 × 10^–23^	1.76 × 10^–23^	1.63 × 10^–23^
r151	C + C_2_O_3_ → O + C_3_O_2_ (−284.4)	3.07 × 10^–25^	5.19 × 10^–23^	2.03 × 10^–24^
r222	CO + C_2_O_2_ → O + C_3_O_2_ (243.7)	1.44 × 10^–25^	4.05 × 10^–23^	1.38 × 10^–24^
r228	C_2_O + CO_2_ → O + C_3_O_2_ (160.8)	3.98 × 10^–24^	4.30 × 10^–23^	3.81 × 10^–25^
r300	CO + C_4_O → C_2_ + C_3_O_2_ (259.1)	1.13 × 10^–24^	1.88 × 10^–23^	1.37 × 10^–23^
r364	CO_2_ + C_3_O → CO + C_3_O_2_ (−152.7)	6.61 × 10^–24^	8.51 × 10^–24^	1.14 × 10^–23^
r365	CO + C_2_O_3_ → O_2_ + C_3_O_2_ (294.0)	1.73 × 10^–23^	1.73 × 10^–24^	3.21 × 10^–27^
r416	CO_2_ + C_2_O_2_ → O_2_ + C_3_O_2_ (276.2)	3.65 × 10^–25^	2.15 × 10^–24^	4.62 × 10^–26^

main C_3_O_2_ consumption reaction routes^a^		pure CO ice (10 K)^b^	CO/CO_2_ (3.9:1) ice (10 K)^b^	pure CO_2_ ice (10 K)^b^
r27	C_3_O_2_ + *R* → C + C_2_O_2_ (Δ*H*_*R*,gas,0K_ = 828.3)	2.23 × 10^–2^	6.34 × 10^–2^	2.79 × 10^–1^
r47	C_3_O_2_ + *R* → O + C_3_O (678.9)	1.58 × 10^–1^	1.38 × 10^–1^	2.26 × 10^–1^
r61	C_3_O_2_ + *R* → C_2_ + CO_2_ (529.2)	3.63 × 10^–2^	4.34 × 10^–1^	4.43 × 10^–2^
r77	C_3_O_2_ + *R* → CO + C_2_O (365.3)	5.69 × 10^–1^	4.98 × 10^–1^	1.05 × 10^–1^
r248	CO + C_3_O_2_ →O + C_4_O_2_ (616.4)	2.55 × 10^–24^	1.07 × 10^–23^	3.40 × 10^–23^
r306	CO + C_3_O_2_ → C_2_ + C_2_O_3_ (753.9)	2.44 × 10^–24^	3.59 × 10^–23^	1.03 × 10^–23^
r357	CO + C_3_O_2_ → O_2_ + C_4_O (788.8)	9.42 × 10^–25^	3.18 × 10^–23^	7.88 × 10^–24^
r361	CO + C_3_O_2_ → C_2_O + C_2_O_2_ (607.8)	1.36 × 10^–24^	1.85 × 10^–23^	1.22 × 10^–23^
r494	CO_2_ + C_3_O_2_ → C_2_O + C_2_O_3_ (590.0)	2.14 × 10^–24^	2.44 × 10^–24^	3.44 × 10^–24^
r525	CO_2_ + C_3_O_2_ → C_2_O_2_ + C_2_O_2_ (524.9)	1.16 × 10^–24^	3.02 × 10^–23^	5.13 × 10^–24^

a Approximate 0 K gas–phase reaction enthalpies (Δ*H*_*R*,gas*,*0K_, in kJ mol^–1^) are also reported. The complete reaction network (642 reactions) and the full set of ERCs are provided in ref 20.

b Units. Radiation-induced dissociation reactions: s^–1^. Bimolecular reactions: cm^3^ molecule^–1^ s^–1^.

Table 1 summarizes the primary production and consumption routes of C_3_O_2_ considered by the PROCODA model in this study, including approximate 0 K gas-phase reaction enthalpies and their labels (e.g., r33, r62). “*R*” indicates cosmic-ray irradiation or its induced secondary electrons within the ice. These routes, also shown in the histograms of Figure 2, constitute a subset of the 642 coupled reactions implemented in the kinetic network.^20^ Notably, all consumption routes are highly endothermic in the gas phase. In irradiated ices, however, energy deposited by incoming cosmic-ray projectiles and their induced secondary electrons readily drives endothermic channels that would otherwise be inaccessible under purely thermal conditions.

It is also important to note that solid-phase (ice) reaction enthalpies can differ substantially from the gas-phase values reported in Table 1, and are difficult to determine experimentally or computationally. The local molecular environment, i.e., matrix effects, evolves during ice irradiation and can strongly modulate the effective reaction enthalpy. For a more in-depth discussion, see ref 19.

Figure 3 presents the evolution of ERCs for selected production (a) and consumption (b) reactions of C_3_O_2_ as a function of the initial CO content in the ice. Reaction labels are indicated (see Table 1), and variations in the ERCs reflect changes in the local chemical environment, with particularly large effects observed for the production reactions. From panel a, we observe that most of the selected ERCs for the production of C_3_O_2_ increase as the CO content decreases—for example, C_4_O_2_ + *R* → C + C_3_O_2_ (r33). However, some reactions show the opposite behavior, such as CO + C_2_O_3_ → O_2_ + C_3_O_2_ (r365), while others appear insensitive to CO abundance, including CO_2_ + C_3_O → CO + C_3_O_2_ (r364). From panel b, we notice that for most of the consumption reactions, ERC values also increase as the CO content decreases, with the exception of C_3_O_2_ + *R* → CO + C_2_O (r77). Again, some reactions display little dependence on CO fraction, C_3_O_2_ + *R* → O + C_3_O (r47) and CO_2_ + C_3_O_2_ → C_2_O + C_2_O_3_ (r494). Finally, in both production and consumption cases, there are reactions that exhibit peculiar or nonmonotonic variations with CO concentration, for instance C_2_O + CO_2_ → O + C_3_O_2_ (r228) and C_3_O_2_ + *R* → C_2_ + CO_2_ (r61).

**Figure 3:**
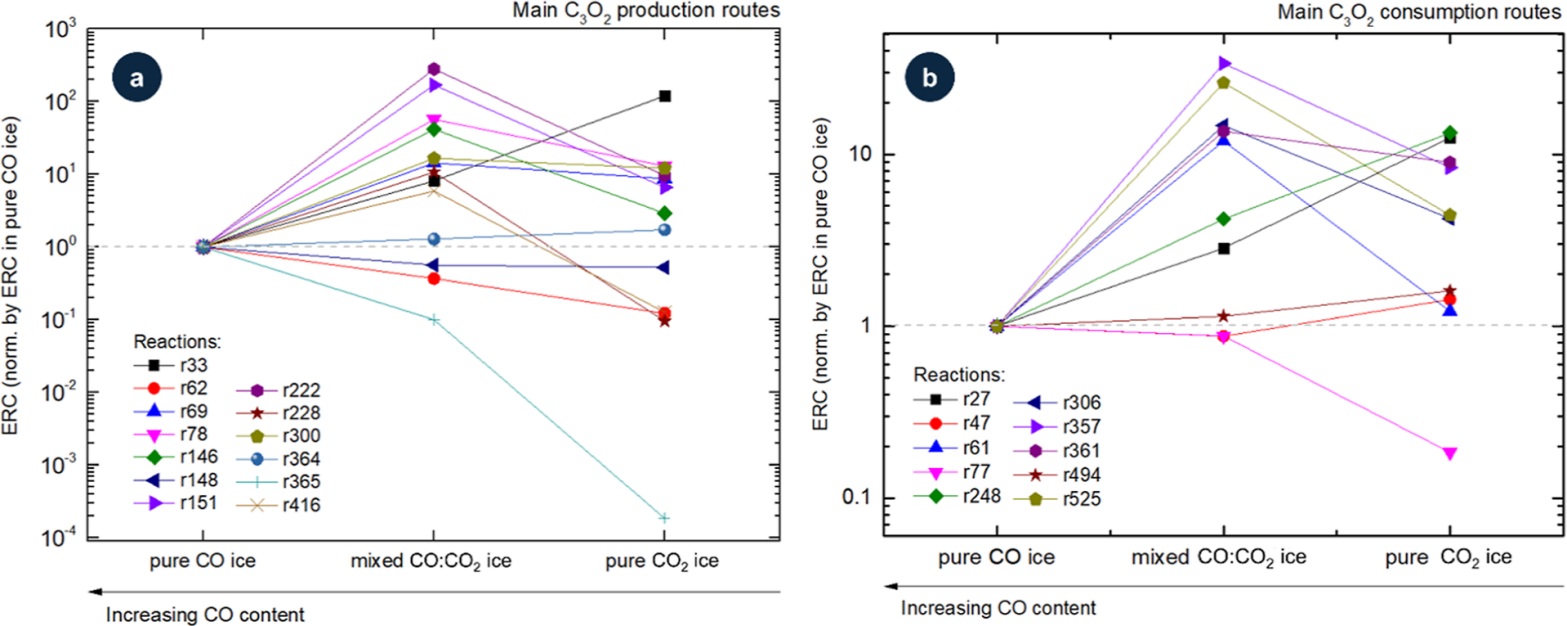
Evolution of ERCs for selected production (a) and consumption (b) reactions of C_3_O_2_ as a function of initial CO content in ices at 10 K. Reaction labels are indicated (see Table 1). Variations in ERCs reflect changes in the local chemical environment.

A combined analysis of both previous works^19,20^ indicates that, while C_3_O_2_ is an important radiolysis product in CO-rich ices, its abundance in mixed CO/CO_2_ and pure CO_2_ ices is negligible (<10^–6^ relative to the initial ice abundance). Furthermore, the current model predicts C_3_O_2_ in pure CO ice at levels roughly an order of magnitude lower than measured in irradiation experiments. This discrepancy may arise from an incomplete reaction network and/or systematic biases in the experimental column densities—for example, uncertainties in baseline treatment and the use of potentially overestimated band strengths taken from prior studies.

#### C_3_O_2_ Production Pathways Evolution in Ices with Different CO Abundances

As shown in Figure 2a, at early irradiation times (*t* = 7 s), the leading production route for C_3_O_2_ is CO + C_2_O → C_3_O_2_ (r78). This exothermic channel (see Table 1) benefits from the relatively high abundance of CO and the build-up of small C–O species under cosmic-ray processing. Another important early pathway is CO + C_3_O → C + C_3_O_2_ (r146), whose contribution is sustained by an early time reservoir of C_3_O generated under energetic processing.^14^ By *t* = 1380 s and into the CE phase (*t* > 1500 s), these two pathways remain among the main contributors, although the dominant channels shift to C_3_ + CO_2_ → C + C_3_O_2_ (r148) and CO + C_2_O_3_ → O_2_ + C_3_O_2_ (r365) as larger species (e.g., C_3_ and C_2_O_3_) accumulate. Interestingly, C_2_O_3_ is likely short-lived in the neutral manifold: Peppe et al.^35^ indicate formation of a transient triplet O_2_C–CO via vertical oxidation of the [O_2_C–CO]^−·^ radical anion, with submicrosecond lifetimes and dissociation channels to CO and CO_2_. In this context, our kinetic treatment regards C_2_O_3_ as a reactive intermediate whose effective abundance and reactivity are captured through the fitted ERCs. Overall, the dominant channels require a balanced supply of small carbon oxides within a CO-rich matrix that acts both as a reactant and an energy moderator (see also refs 15,36 ), and the model behavior is consistent with ion-irradiation experiments on CO ice that report efficient C_3_O_2_ formation via stepwise association processes.^4^

For the mixed CO/CO_2_ (3.9:1) ice (Figure 2c), early irradiation (*t* ≲ 500 s) still favors CO + C_2_O → C_3_O_2_ (r78), followed by CO + C_2_O_2_ → O + C_3_O_2_ (r222), but the presence of CO_2_ enables additional routes such as and C_2_O + CO_2_ → O + C_3_O_2_ (r228). In the CE phase, these same three routes dominate, albeit with slightly shifting relative contributions as the system re-equilibrates, in comparison with the pattern observed in the small fluence domain. The model therefore indicates that even modest amounts of CO_2_ open additional, energetically accessible formation channels for C_3_O_2_—consistent with experimental observations that intermediate CO/CO_2_ mixtures often yield higher steady-state abundances of C_3_O_2_.^14,19^ A key point here is that CO_2_ can efficiently generate C_2_O_2_ and related carbon–oxide intermediates under energetic bombardment.^15^ These transient species supplement the typical CO + C_*x*_O_*y*_ channels and thus broaden the C_3_O_2_ formation network (see also ref 16). As cosmic rays continuously produce reactive fragment and electronically excited species, the mixed ice sustains multiple complementary pathways to assemble the C_3_O_2_ skeleton.

The dominant production routes for C_3_O_2_ in irradiated pure CO_2_ ice are presented in Figure 2e. At low fluence, formation proceeds chiefly via CO_2_ + C_2_O_2_ → O_2_ + C_3_O_2_ (r416), followed by C_2_ + CO_2_ → C_3_O_2_ (r62) and CO_2_ + C_3_O → CO + C_3_O_2_ (r364). Notably, C_2_ and C_2_O_2_ species are generated by dissociation processes induced by cosmic rays in the CO_2_ ice.^26^ By the time the system reaches chemical equilibrium (*t* > 1500 s), CO + C_2_O_2_ → O + C_3_O_2_ (r222) and C_3_ + CO_2_ → C + C_3_O_2_ (r148) become more prevalent, reflecting the slow buildup of CO and C_3_ under continued irradiation (see also ref 20). From an astrochemical perspective, the production of C_3_O_2_ in a pure CO_2_ environment underscores the robust capacity of high-energy particles to dissociate and rearrange CO_2_-rich ices into extended carbon oxides (C_*x*_O_*y*_). Although less straightforward than in CO-rich ices, these results highlight that even relatively “oxidized” matrices can still yield carbon suboxide, especially at advanced fluences.

#### C_3_O_2_ Consumption Pathways in Ices with Different CO Abundances

Figure 2b shows that, in pure CO ice, C_3_O_2_ is consumed mainly through radiation-driven channels across both low- and high-fluence regimes. We denote these processes by *R*, where *R* captures interactions with either the incident projectile or the secondary electrons it generates in the ice. The leading pathway is the direct fragmentations C_3_O_2_ + *R* → CO + C_2_O (r77) and C_3_O_2_ + *R* → O + C_3_O (r47). The matrix also participates chemically: a recurrent bimolecular sink, typically third or fourth in rank, is CO + C_3_O_2_ → O + C_4_O_2_ (r248).

For the CO/CO_2_ mixture (Figure 2d), C_3_O_2_ + *R* → CO + C_2_O (r77) consistently emerges as the leading destruction channel across all time points, paralleling the behavior in pure CO ice. However, for small fluences, competition with the reaction CO + C_3_O_2_ → C_2_ + C_2_O_3_ (r306) is important. By contrast, the CO_2_ + C_3_O_2_ collision channel is negligible in this mixture, indicating that, despite the presence of both parent species, the principal collisional sink is governed by CO, plausibly reflecting its greater mobility in the matrix at 10 K. Overall, adding CO_2_ to a CO matrix broadens the set of accessible loss pathways but does not alter the primary radiation-induced consumption mechanism.

For pure CO_2_ irradiation (Figure 2f), the dominant C_3_O_2_ loss channels differ from those in pure CO and mixed CO/CO_2_ ices. The leading routes are radiation-driven fragmentations C_3_O_2_ + *R* → C + C_2_O_2_ (r27) and C_3_O_2_ + *R* → O + C_3_O (r47). Important collisional pathways with the matrix are CO_2_ + C_3_O_2_ → C_2_O + C_2_O_3_ (r494) and CO_2_ + C_3_O_2_ → 2 C_2_O_2_ (r525). The observed competition between r27 and r47 highlights how cosmic-ray-induced dissociation can abstract different atoms from the carbonyl termini, setting distinct branching ratios.^13^ These trends accord with prior irradiation experiments showing that, in CO_2_-rich matrices, carbon suboxide is prone to cleavage at the outer carbonyl groups, yielding C_3_O or C_2_O_2._ ^26^

Figure 4 provides a schematic overview of how the dominant pathways shift between the small-fluence regime and the CE phase (*t* > 1500 s), and how these patterns differ among pure CO, mixed CO/CO_2_, and pure CO_2_ ices. The side-by-side panels trace the evolution of the C_3_O_2_ reaction network as the ice transitions from early irradiation—when reactive intermediates first accumulate and seed initial production—to the CE phase, where multiple competing channels attain quasi-steady behavior. In pure CO ice, the dominant routes change with fluence: at low fluence, production is dictated by bimolecular collisions involving CO, whereas by the CE phase a CO_2_-involving channel becomes a principal contributor (C_3_ + CO_2_ → C + C_3_O_2_, r148). In the mixed CO/CO_2_ ice, the ranking of dominant pathways remains largely stable across fluence. By contrast, in pure CO_2_ ice, the dominant production routes vary markedly: early on, formation is driven by bimolecular collision with CO_2_ (CO_2_ + C_2_O_2_ → O_2_ + C_3_O_2_; r416), but at CE this is supplanted by bimolecular collision with CO (CO + C_2_O_2_ → O + C_3_O_2_; r222).

**Figure 4:**
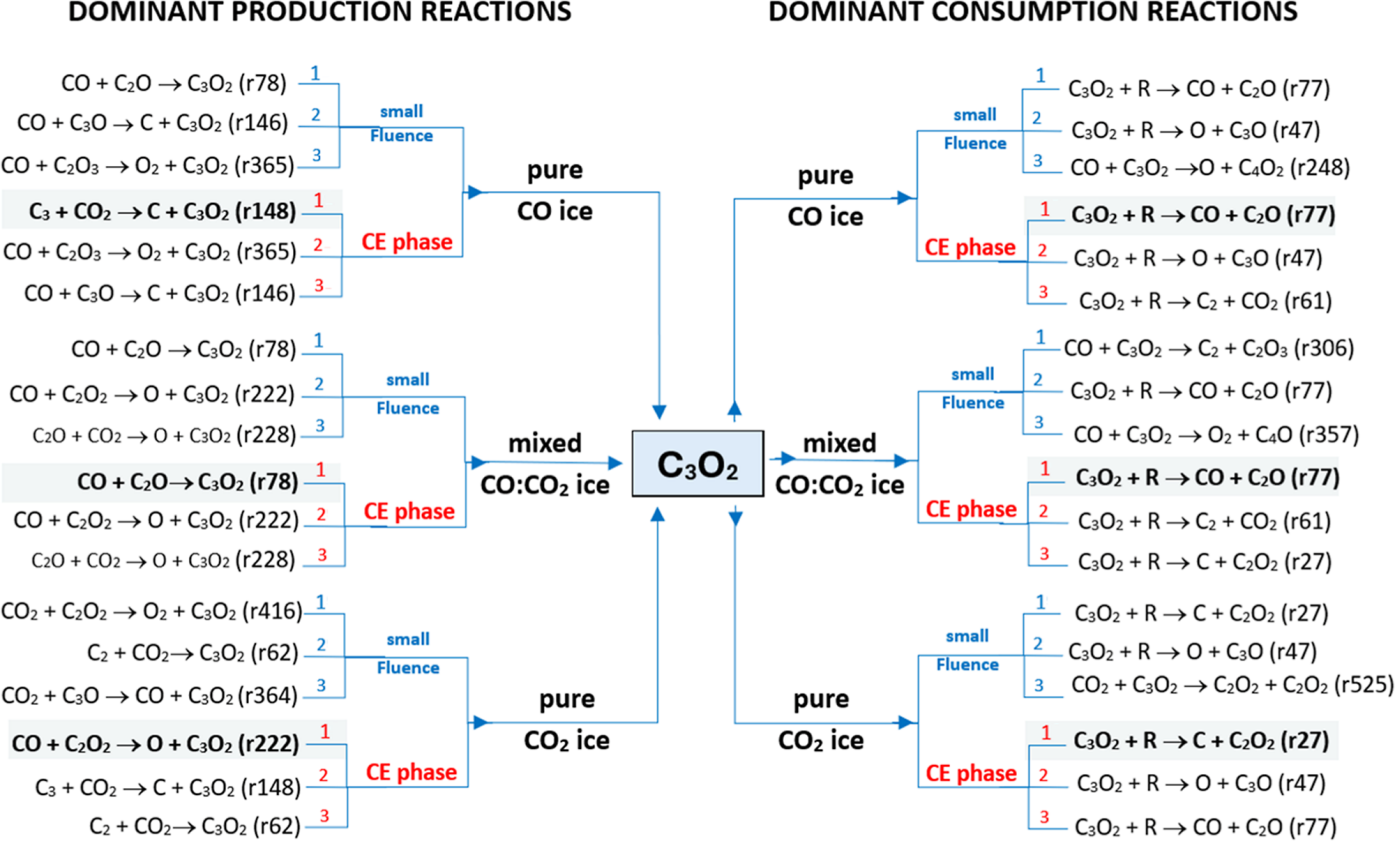
Schematic illustration of the main production and consumption routes of C_3_O_2_ as a function of initial CO/CO_2_ abundances in ices irradiated by cosmic-ray analogs. Hatched arrows denote the dominant pathways in the chemical-equilibrium (CE) phase. Numbers 1, 2, and 3 indicate the primary, secondary, and tertiary dominant reaction pathways, respectively. See text for details.

The primary destruction mechanism for C_3_O_2_ in all simulated ices is radiation-induced dissociation, though the specific dominant pathways and products are highly dependent on the ice matrix. In pure CO ice, direct dissociation to CO + C_2_O (r77) rapidly becomes the key sink as the radical population grows, ultimately prevailing at CE. This finding is consistent with the experimental results of Bennet et al.^26^ employing 5 keV electrons in CO ice. At low fluences, however, this channel is temporarily rivaled by pathways like CO + C_3_O_2_ → O + C_4_O_2_ (r248). In the mixed CO/CO_2_ ice, other routes emerge at early times (e.g., CO + C_3_O_2_ → C_2_ + C_2_O_3_; r306), but direct dissociation again dominates the consumption of C_3_O_2_ at CE. Finally, in pure CO_2_ ice, consumption is characterized by outer-carbonyl cleavage, primarily through the removal of a carbon atom (C_3_O_2_ + *R* → C + C_2_O_2_; r27) or an oxygen atom (C_3_O_2_ + *R* → O + C_3_O; r47), with r27 prevailing at CE. Collectively, these results show that while radiation-mediated bond breaking is the principal sink for C_3_O_2_, the matrix controls the specific fragmentation products (e.g., C_2_O vs C_2_O_2_), thereby controlling the subsequent chemistry in astrophysical ices.

These contrasting patterns among pure CO, mixed CO/CO_2_, and pure CO_2_ ices in Figure 3 highlight the context-dependence of C_3_O_2_ chemistry under irradiation and identify the key primary, secondary, and tertiary reaction channels needed to refine astrochemical models of irradiated ices. ERC values for all discussed reactions are listed in ref 20.

### Influence of Ice Temperature in Reaction Routes of C_3_O_2_ in Pure CO during Irradiation by Cosmic Rays

In order to investigate the role of temperature on the kinetics of C_3_O_2_ formation and consumption, we extended the analysis of pure CO ice irradiation (see Figure 2) also to 15 and 20 K. Figure 5 shows the time-dependent contributions of the seven most dominant reaction pathways (both production and consumption) of C_3_O_2_ under these two new temperature conditions. Similarly to the 10 K case, the early irradiation regime (*t* ≲ 100 s) is characterized by a variety of bimolecular collisions leading to the formation of small species (C, C_2_, C_2_O, among others), which then recombine or rearrange to form C_3_O_2_. In each figure, panel (a) shows the dominant production pathways, and panel (b) shows the dominant consumption pathways, with labeled reactions detailed in Table 2.

**Figure 5:**
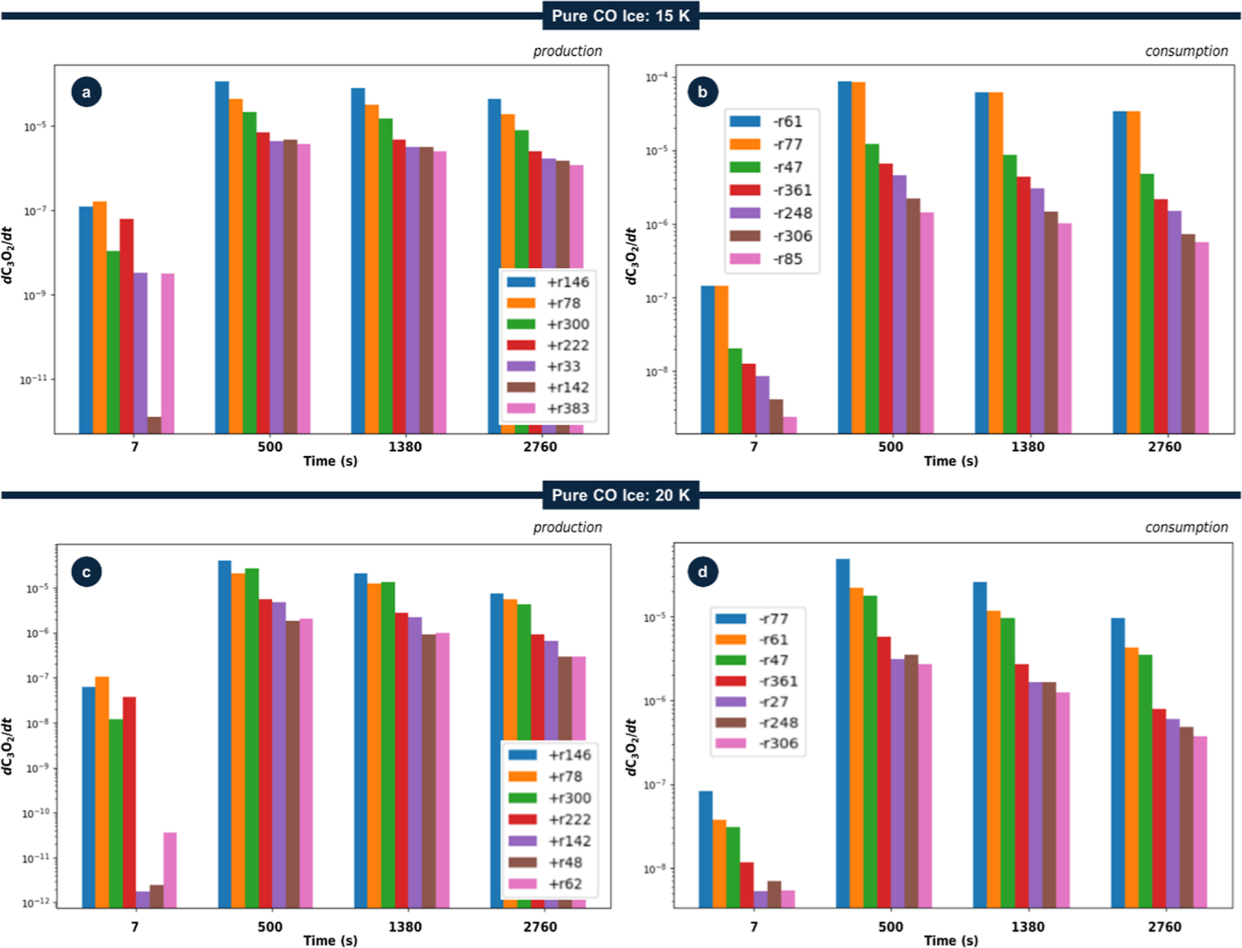
Time evolution of the contributions of the seven dominant reaction pathways to the production (a,c) and consumption (b,d) of C_3_O_2_ in pure CO ice irradiated by cosmic rays at (a,b) 15 K and (c,d) 20 K. The *y*-axis shows the differential production/consumption rate (divided by the largest column density of the parent species at time = 0 s) evaluated at the times indicated on the *x*-axis. See the main text for details.

**Table 2:** Main Production and Consumption Routes for C_3_O_2_ and Their Corresponding ERCs Used in the Model (Corresponding to Figures 2 and 5) for Ices at 10 K

main C_3_O_2_ production reaction routes^a^		pure CO ice (10 K)*^b^	pure CO ice (15 K)^b^	pure CO ice (20 K)^b^
r33	C_4_O_2_ + *R* → C + C_3_O_2_ (Δ*H*_*R*,gas,0K_ = 455.6)	5.94 × 10^–3^	1.15 × 10^–1^	8.63 × 10^–2^
r62	C_2_ + CO_2_ → C_3_O_2_ (−529. 3)	1.38 × 10^–23^	1.08 × 10^–24^	2.64 × 10^–24^
r69	C_5_O_2_ + *R* → C_2_ + C_3_O_2_ (566.3)	3.56 × 10^–2^	3.78 × 10^–2^	3.07 × 10^–2^
r78	CO + C_2_O → C_3_O_2_ (−365.3)	1.00 × 10^–24^	9.67 × 10^–24^	6.27 × 10^–24^
r146	CO + C_3_O → C + C_3_O_2_ (393.2)	4.15 × 10^–25^	6.37 × 10^–24^	2.73 × 10^–24^
r148	C_3_ + CO_2_ → C + C_3_O_2_ (187.8)	3.11 × 10^–23^	3.39 × 10^–24^	2.84 × 10^–24^
r151	C + C_2_O_3_ → O + C_3_O_2_ (−284.4)	3.07 × 10^–25^	1.39 × 10^–23^	8.22 × 10^–24^
r222	CO + C_2_O_2_ → O + C_3_O_2_ (243.7)	1.44 × 10^–25^	3.23 × 10^–24^	3.06 × 10^–24^
r228	C_2_O + CO_2_ → O + C_3_O_2_ (160.8)	3.98 × 10^–24^	5.79 × 10^–24^	4.32 × 10^–24^
r300	CO + C_4_O → C_2_ + C_3_O_2_ (259.1)	1.13 × 10^–24^	5.31 × 10^–24^	7.44 × 10^–24^
r364	CO_2_ + C_3_O → CO + C_3_O_2_ (−152.7)	6.61 × 10^–24^	1.26 × 10^–24^	1.24 × 10^–24^
r365	CO + C_2_O_3_ → O_2_ + C_3_O_2_ (294.0)	1.73 × 10^–23^	8.54 × 10^–25^	1.13 × 10^–24^
r416	CO_2_ + C_2_O_2_ → O_2_ + C_3_O_2_ (276.2)	3.65 × 10^–25^	3.05 × 10^–24^	3.22 × 10^–24^

main C_3_O_2_ consumption reaction routes^a^		pure CO ice (10 K)*^b^	pure CO ice (15 K)^b^	pure CO ice (20 K)^b^
r27	C_3_O_2_ + *R* → C + C_2_O_2_ (Δ*H*_*R*,gas,0K_ = 828.3)	2.23 × 10^–2^	1.16 × 10^–2^	2.98 × 10^–2^
r47	C_3_O_2_ + *R* → O + C_3_O (678.9)	1.58 × 10^–1^	1.11 × 10^–1^	1.73 × 10^–1^
r61	C_3_O_2_ + *R* → C_2_ + CO_2_ (529.2)	3.63 × 10^–2^	7.86 × 10^–1^	2.12 × 10^–1^
r77	C_3_O_2_ + *R* → CO + C_2_O (365.3)	5.69 × 10^–1^	7.83 × 10^–1^	4.68 × 10^–1^
r248	CO + C_3_O_2_ →O + C_4_O_2_ (616.4)	2.55 × 10^–24^	2.67 × 10^–24^	2.25 × 10^–24^
r306	CO + C_3_O_2_ → C_2_ + C_2_O_3_ (753.9)	2.44 × 10^–24^	1.28 × 10^–24^	1.72 × 10^–24^
r357	CO + C_3_O_2_ → O_2_ + C_4_O (788.8)	9.42 × 10^–25^	6.00 × 10^–25^	4.08 × 10^–25^
r361	CO + C_3_O_2_ → C_2_O + C_2_O_2_ (607.8)	1.36 × 10^–24^	3.85 × 10^–24^	3.70 × 10^–24^
r494	CO_2_ + C_3_O_2_ → C_2_O + C_2_O_3_ (590.0)	2.14 × 10^–24^	1.69 × 10^–24^	1.51 × 10^–24^
r525	CO_2_ + C_3_O_2_ → C_2_O_2_ + C_2_O_2_ (524.9)	1.16 × 10^–24^	6.80 × 10^–24^	1.09 × 10^–23^

a Δ*H*_*R,gas,*0K_ values (in kJ mol^–1^) are also reported. The Complete Reaction Network (642 Reactions) and the Full Set of ERCs are provided in Ref 20.[Table-fn t2fn1]

b Units. Radiation-induced dissociation reactions: s^–1^. Bimolecular reactions: cm^3^ molecule^–1^ s^–1^. * Same values from Table 1.

Table 2 lists the key production and consumption routes for C_3_O_2_ in pure CO ices at 10, 15, and 20 K, together with their respective ERCs and approximate 0 K gas-phase reaction enthalpies. This table shows that radiation-driven pathways exhibit smaller relative temperature dependence than bimolecular routes. This likely arises because radiation-induced processes are driven primarily by energy deposition from cosmic-ray analogs rather than by thermally activated diffusion or collisions. In contrast, several bimolecular reactions, e.g., CO + C_4_O → C_2_ + C_3_O_2_ (r300) and C + C_2_O_3_ → O + C_3_O_2_ (r151), display increases or decreases in ERC with temperature, reflecting temperature-sensitive mobility and encounter rates within the CO matrix.

By systematically comparing these ERC values at 10, 15, and 20 K, Table 2 highlights the nuanced interplay between thermal effects, the abundances of reactive intermediates, and the local chemical environment. These trends are particularly relevant to the cold regions of protoplanetary disks and dense molecular clouds, where slight temperature variations (on the order of a few Kelvin) can shift the balance among competing pathways and thereby modify the net formation rate of complex species like C_3_O_2_ (see also refs 4,26 ).

Figure 6 illustrates how ERCs for selected production and consumption reactions of C_3_O_2_ vary with temperature in pure CO ice. Specifically, it compares the behavior at 10, 15, and 20 K for several reaction pathways already identified as key contributors to C_3_O_2_ chemistry. The values were normalized to the 10 K ERCs to emphasize relative changes and reduce the impact of systematic offsets.

**Figure 6:**
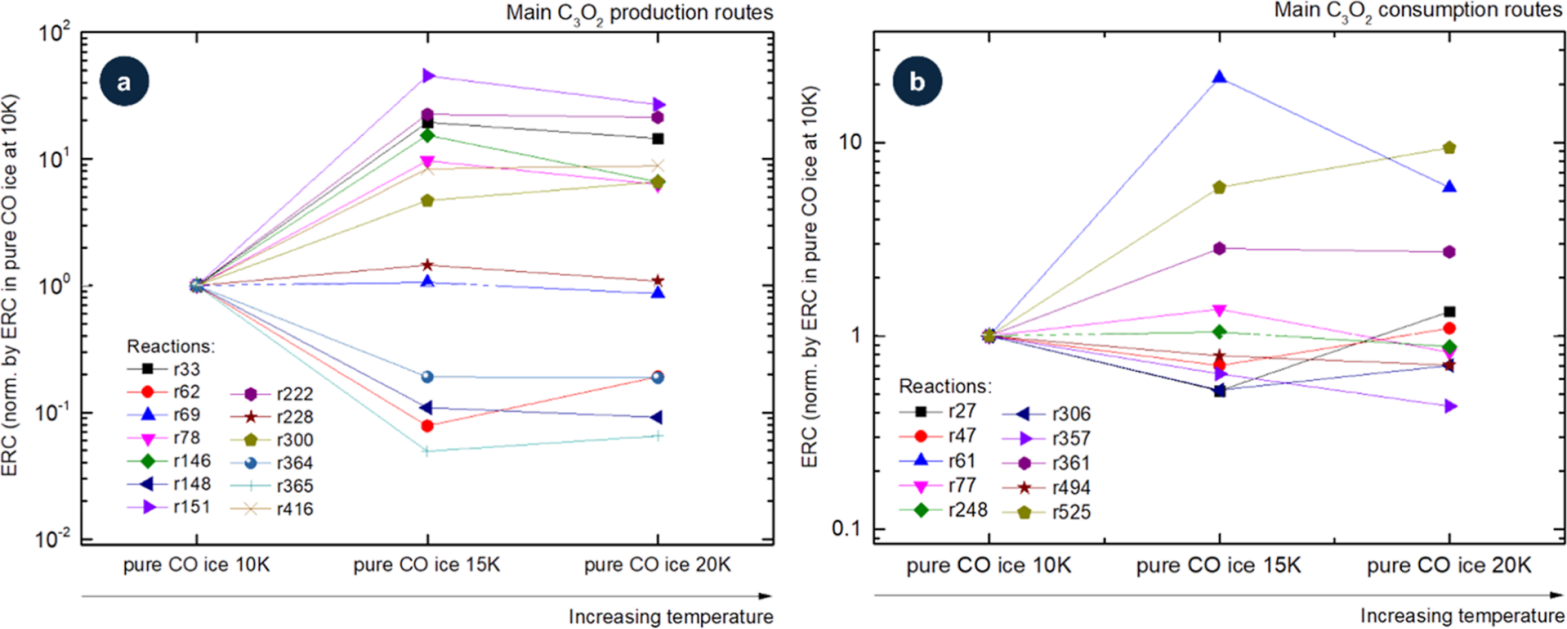
Evolution of ERCs for selected production (a) and consumption (b) reactions of C_3_O_2_ as a function of the temperature. Reaction labels are indicated (see Table 2). Variations in ERCs reflect changes in the local chemical environment.

On the production side (Figure 6a), several bimolecular channels show modest ERC increases at higher temperature—consistent with small thermal enhancements to molecular mobility (e.g., C_5_O_2_ + *R* → C_2_ + C_3_O_2_, r69; C + C_2_O_3_ →O + C_3_O_2_, r151). By contrast, some routes decrease with temperature (e.g., C_3_ + CO_2_ → C + C_3_O_2_, r148; CO_2_ + C_3_O → CO + C_3_O_2_, R364). Temperature-dependent pathways may contribute more significantly at 20 K than at 10 K, particularly at higher fluences when transient intermediates accumulate.^15,19^ For consumption (Figure 6b), radiation-induced pathways remain dominant across temperatures, mainly C_3_O_2_ + *R* → CO + C_2_O (r77) in competition with C_3_O_2_ + *R* → C_2_ + CO_2_ (r61) or C_3_O_2_ + *R* → O + C_3_O (r47), depending on the temperature. Although the ordering among consumption routes changes only slightly, individual ERCs do vary with temperature (see Table 2). Notably, several reactions (e.g., C_2_O + CO_2_ → O + C_3_O_2_, r228; C_5_O_2_ + *R* → C_2_ + C_3_O_2_, r69; and CO + C_3_O_2_ → O + C_4_O_2_, r248) are essentially insensitive to the 10–20 K range.

From an astrophysical perspective, these results indicate that local ice temperatures typical of dense clouds and protoplanetary disks (∼10–30 K) can critically modulate both formation and destruction of complex organic precursors (herein exemplified by C_3_O_2_) under cosmic-ray bombardment. Therefore, incorporating such temperature dependences into astrochemical models is crucial for accurately simulating ice composition gradients in star-forming regions (see also refs 6,17 ).

#### C_3_O_2_ Production Pathways Evolution in CO Ices with Different Temperatures

A direct comparison of Figures 2a and 5a,c reveals that, while the core set of production reactions for C_3_O_2_ in pure CO ice remains consistent (e.g., CO + C_2_O → C_3_O_2_, r78; or C_3_ + CO_2_ → C + C_3_O_2_, r148), their relative importance shifts with temperature. At 10 K (Figure 2a), early time formation is dominated by CO + C_2_O → C_3_O_2_ (r78), while at the CE phase C_3_ + CO_2_ → C + C_3_O_2_ (r148) becomes dominant. However, at 15 K and above, CO + C_3_O → C + C_3_O_2_ (r146) takes over as the principal formation channel. In addition, increased molecular mobility at higher temperatures brings other routes, such as CO + C_4_O → C_2_ + C_3_O_2_ (r300), into greater prominence (see Table 2 for numerical values).

The results show that specific channels gain or lose relative weight as temperature increases and diffusion of reactive intermediates becomes more efficient. In particular, reactions with modest activation barriers, such as CO + C_2_O → C_3_O_2_ (r78) and CO + C_4_O → C_2_ + C_3_O_2_ (r300), become comparatively more effective at 15 and 20 K than at 10 K, reflecting the slight enhancement of mobility and encounter rates within the ice matrix (see also refs 16 and 19 ).

#### C_3_O_2_ Consumption Pathways Evolution in CO Ices with Different Temperatures

Figures 2b and 5b,d show the main consumption routes for C_3_O_2_ as a function of irradiation time for three ices with different temperatures. Across all temperatures, radiation-induced dissociation (e.g., C_3_O_2_ + *R* → CO + C_2_O, r77) remains a principal sink, with additional competition from bimolecular pathways—particularly at longer irradiation times as reactive intermediates accumulate. Overall, these results reinforce that even modest temperature increases within 10–20 K can reshape both the production and destruction networks of C_3_O_2_ in CO ices. Quantifying these variations is essential for refining astrochemical models of dense cloud interiors and early protostellar environments, where small temperature gradients may influence the emergence of prebiotic molecules.

Overall, the temperature dependence of ERCs shows that radiation-driven pathways (e.g., C_3_O_2_ + *R* → C + C_2_O_2_, r27) change little in absolute rate between 10 and 20 K, whereas several bimolecular channels display a markedly stronger temperature sensitivity. These involve reactive intermediates that must diffuse through the ice to encounter C_3_O_2_ or one another (e.g., C_3_O, C_2_O_2_, C_2_O_3_). As temperature increases from 10 to 20 K, enhanced mobility raises encounter probabilities and thus the associated reaction rates.^14,15^ Nevertheless, at high fluence, where the ice approaches chemical equilibrium, many pathways converge to similar relative weights, implying that across a 10 K span, temperature alone does not drastically reset the steady-state composition given sustained ionizing flux. This subtle but consequential temperature effect should be captured in astrochemical models, particularly for protoplanetary disks where Kelvin-scale radial gradients can imprint spatially dependent reaction networks and, hence, spatial variations in species such as C_3_O_2_ (see also refs 1 and 28 ).

Figure 7 schematically compares the most important C_3_O_2_ formation and consumption pathways in pure CO ices at the three temperatures investigated. The figure highlights how the interplay between the availability of reactive intermediates, the single-component CO matrix, and modest thermal activation reshapes pathway rankings from the low-fluence (early irradiation) regime to the CE phase at higher fluence. Early time production at all three temperatures is often dictated by CO + C_2_O → C_3_O_2_ (r78), whereas at CE the dominant formation route shifts to C_3_ + CO_2_ → C + C_3_O_2_ (r148) at 10 K and to CO + C_3_O → C + C_3_O_2_ (r146) at *T* ≥ 15 K. On the consumption side, qualitative changes with temperature are minor: at CE the dominant pathway is almost always C_3_O_2_ + *R* → CO + C_2_O (r77). Temperature-dependent differences in the magnitudes of ERCs are, however, evident and are reported in Table 2.

**Figure 7:**
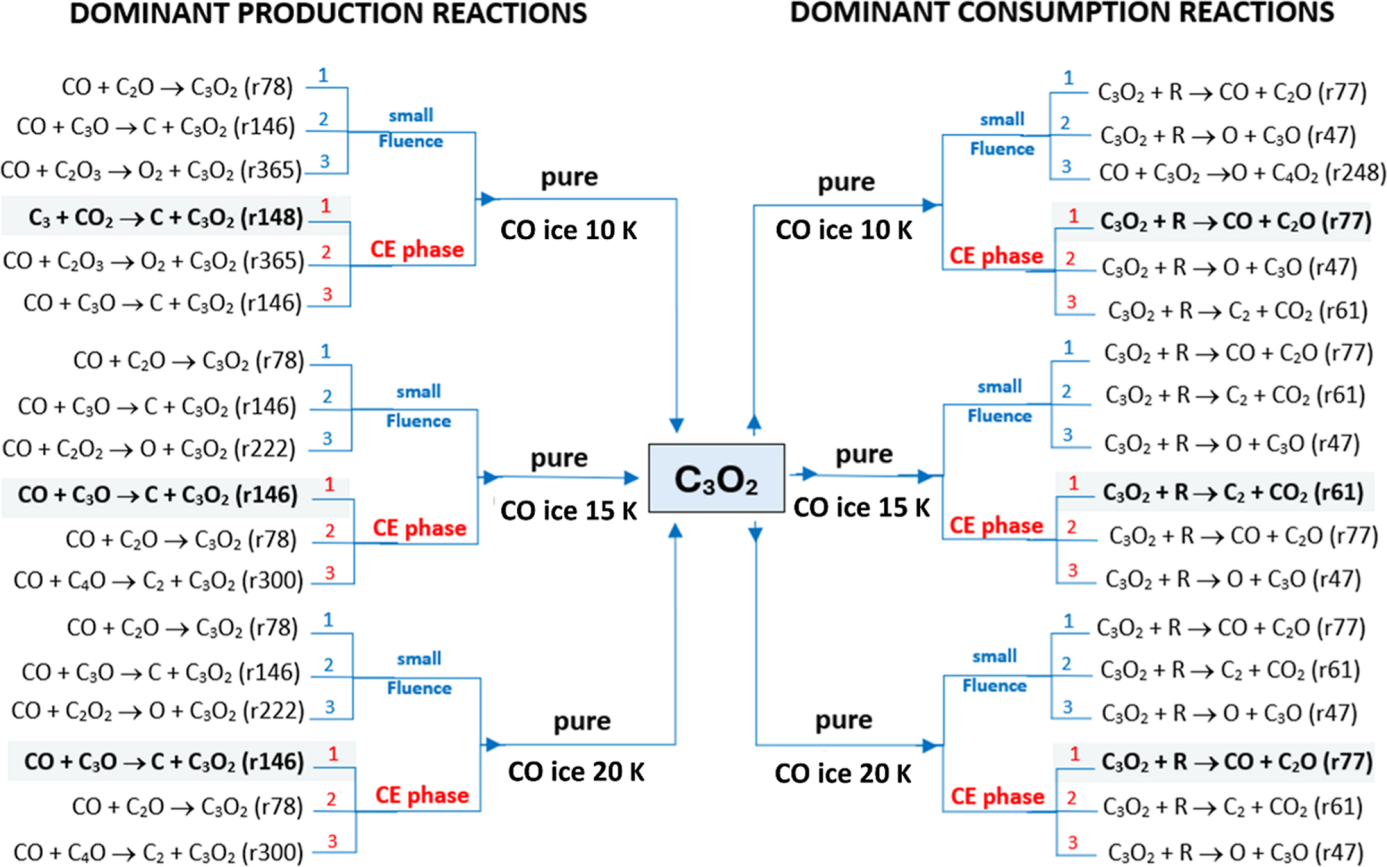
Schematic illustration of the main production and consumption routes of C_3_O_2_ as a function of ice temperatures (10, 15, and 20 K) in pure CO ices irradiated by cosmic-ray analogs. Hatched arrows denote the dominant pathways in the chemical-equilibrium (CE) phase. Numbers 1, 2, and 3 indicate the primary, secondary, and tertiary dominant reaction pathways, respectively. See text for details.

In the early stages of irradiation (*t* < 100 s), the generation of reactive intermediates is driven primarily by the cosmic-ray flux, with temperature acting as a secondary control on mobility. Once their densities become sufficiently high, small temperature differences can produce markedly different encounter efficiencies—especially for heavier intermediates—so routes involving species such as C_4_O, C_3_O, or C_2_O_2_ can show temperature-enhanced branching even though the energetic driver is unchanged. Over longer irradiation times, all temperatures trend toward equilibrium-like behavior, implying that the eventual product distribution is constrained mainly by the total deposited energy, while temperature modulates the rate at which the system approaches that state (e.g., refs 5 and 6 ).

From a physicochemical standpoint, temperature-dependent ERC variations—and the associated shifts in dominant routes—arise from four complementary factors: (i) cosmic-ray-induced dissociation, which continuously generates reactive intermediates largely independent of temperature; (ii) incremental mobility of these intermediates, which becomes non-negligible as temperature rises; (iii) temperature-driven changes in the local chemical environment (matrix structure, composition, and trapping); and (iv) the explicit temperature dependence of the rate coefficients themselves. Together, these effects shape the abundance and lifetime of carbon suboxide in CO ices, underscoring the strong environmental control on its fate (see also refs 6,26 ).

### Astrochemical Implications

Astrochemical ices, particularly those rich in CO and CO_2_, are subject to evolving reaction networks under ionizing radiation, where even subtle temperature differences (10–20 K) and varying exposure times can strongly modulate the formation of key species like C_3_O_2_. In colder regions (*T* ≤ 10 K), typical of dense molecular cloud cores, slower diffusion limits C_3_O_2_ production, whereas slightly elevated temperatures (∼20 K) can accelerate its early formation. As the cumulative radiation dose increases and the ice approaches chemical equilibrium (CE), pathway rankings shift, yielding distinct chemistries across protoplanetary disks—near the star versus in dust-obscured zones. Motivated by this environmental sensitivity, we systematically chart how temperature and composition reshape the dominant routes for C_3_O_2_ (and related species) from low-fluence stages to near-equilibrium, refining our picture of ice processing in space.

A primary finding of this work is that the approach to CE in radiation-processed ices plays a pivotal role in astrochemistry, defining how reaction networks evolve under prolonged irradiation and ultimately determining the steady-state abundances of key species. Our results show that reaction pathways leading to molecules such as C_3_O_2_ can shift dramatically as ices transition from low fluence to CE, indicating that both initial composition and cumulative radiation dose must be accounted for when modeling the chemical complexity of dense molecular clouds, protoplanetary disks, and other cold environments.

Another key finding is that the local chemical environment—whether CO- or CO_2_-rich—and the ice temperature (10–20 K) both substantially modify ERCs. At slightly higher temperatures (∼20 K), increased mobility of reactive intermediates accelerates certain bimolecular channels and boosts early stage C_3_O_2_ formation, whereas at lower temperatures (*T* ≤ 10 K) diffusion limits slow its appearance but do not preclude eventual formation under sustained irradiation. These results underscore the need to model composition, temperature, and ionizing flux in concert when identifying dominant reaction routes, rather than assuming uniform chemistry across diverse astrophysical settings.

From an astrochemical modeling perspective, incorporating these environment-dependent ERC variations is crucial for accurately simulating ice chemistry in star-forming regions. Temperature gradients within protoplanetary disks, for instance, can vary by several kelvin over short radial distances. Our findings indicate that small (10 K) changes can reorganize dominant reaction pathways and thus shift the abundances of intermediate and final molecular products. Accounting for these effects in large astrochemical networks will lead to more robust predictions of ice compositions and more realistic interpretations of spectroscopic data from facilities like JWST and ALMA (see also refs 19,26 , and the recent review by Dickers et al.^37^).

Finally, the detectability of reactive species such as C_3_O_2_ in comets, Kuiper Belt objects, and protostellar envelopes may hinge on subtle matrix and temperature effects. On the cold, CO/CO_2_-rich surfaces of outer Solar System bodies—now well documented by JWST^38–41^ —radiation processing can drive the pathways outlined here, potentially contributing to low-albedo mantles and feeding the inventory of more complex organics.^14,42,43^ These considerations argue for treating C_3_O_2_ not merely as a transient radiation product but as a key intermediate in the solid-phase chemistry of cold environments. As observations push to fainter spectral signatures, models that incorporate temperature-sensitive ERCs, matrix composition, and irradiation stage (pre-CE vs near-CE) will be essential for interpreting the data and elucidating the chemistry of cosmic ices.

By using C_3_O_2_ as a prototype molecule, the present research spotlights how changes in both the chemical environment and temperature can markedly shape the reaction dynamics of irradiated astrophysical ices, and future work focusing on other species will further clarify how neighboring molecules within the ice impact these chemical pathways.^44,45^

## Conclusions

This study sheds light on how chemical environment influences reaction dynamics in astrophysical ices under irradiation. We revisited the outputs from the PROCODA kinetic model applied to the previously studied mixed CO/CO_2_ ices^20^ and pure CO at three distinct temperatures^19^ irradiated by cosmic rays with the focus on the production and consumption of carbon suboxide (C_3_O_2_), as a prototype molecule for this investigation. Although C_3_O_2_ has not yet been directly detected in the ISM, it is predicted to play a crucial role in the chemical evolution of astrophysical ices and may significantly influence the broader astrochemical network. The employed model considers 642 coupled chemical equations to map the chemical evolution of 18 species within the ices, including C_3_O_2_, during irradiation by cosmic ray analogs. Our main conclusions are:

(i) Changes in the chemical environment—whether through different neighboring molecules or modest temperature variations—alter the effective rate coefficients (ERCs) for the production and consumption of C_3_O_2_ in irradiated ices.
(ii) Beyond shifts in ERC values, the evolving abundances of reactants during irradiation (up to the CE phase) lead to changes in the preferential pathways for C_3_O_2_ production and destruction. This reinforces the need to understand how the ice environment, together with energetic secondary particles, influences reaction dynamics.
(iii) In the early stages, formation chemistry is dominated by the matrix species: in pure CO ice, CO + C_2_O → C_3_O_2_ (r78) leads; in pure CO_2_ ice, CO_2_ + C_2_O_2_ → O_2_ + C_3_O_2_ (r416) prevails. As irradiation proceeds to CE, the dominant channel shifts: to C_3_ + CO_2_ → C + C_3_O_2_ (r148) in pure CO (as CO_2_ and larger carbon species accumulate) and to CO + C_2_O_2_ → O + C_3_O_2_ (r222) in pure CO_2_ (as CO builds up). In mixed CO/CO_2_ ice, the ranking is comparatively stable from early times to CE, with CO-involving routes remaining the principal contributors.
(iv) At CE, the principal consumption pathway of C_3_O_2_ also depends on the ice composition, though it consistently proceeds through radiation-induced dissociation. In pure CO ice, the dominant route is C_3_O_2_ + *R* → CO + C_2_O (r77), while in pure CO_2_ ice it is C_3_O_2_ + *R* → C + C_2_O_2_ (r27), underscoring again the role of the chemical environment in dictating reaction mechanisms.
(v) ERC values for both formation and destruction pathways also vary with temperature. Some bimolecular reactions increase in rate between 10–20 K, while a smaller subset decreases. These effects, driven largely by enhanced mobility of reactive intermediates, are less pronounced than those arising from changes in ice composition. At high fluences, the system converges to a broadly similar steady-state composition, though subtle thermal influences must still be incorporated into astrochemical models.

Ultimately, the results and discussion presented in this work advance the integration of laboratory data with astrophysical conditions and highlight C_3_O_2_ as a key intermediate in the cosmic synthesis of complex organics and prebiotic molecules in cold, irradiated environments. Using C_3_O_2_ as a prototype, this study demonstrates that both chemical environment and temperature strongly modulate the reaction dynamics of ices under ionizing radiation. Extending this approach to other molecular systems will be essential for refining our understanding of environment-dependent pathways in astrochemical models.

## Data Availability

The data supporting the findings of this study are available within the article and its Supporting Information. Additional raw data sets and intermediate files are available from the corresponding author upon reasonable request, as these materials include large-volume computational outputs that are not practical to host in a public repository. The PROCODA code used in this work is available from the authors for academic, noncommercial use.
